# When Body and Space Shape Perceived Health: Spatial and Interoceptive Processes in Amnestic Mild Cognitive Impairment

**DOI:** 10.3390/jcm15207787

**Published:** 2026-10-09

**Authors:** Sabrina Iuliano, Scila Nunziata, Francesco Ruotolo, Alessandro Iavarone, Tina Iachini, Maria Rosaria Pasciucco, Francesca Ferri, Marcello Costantini, Gennaro Ruggiero

**Affiliations:** 1 Department of Psychology, Università Degli Studi Della Campania “Luigi Vanvitelli”, Viale Ellittico, 31, 81100 Caserta, Italy; sabrina.iuliano@unicampania.it (S.I.); scila.nunziata@unicampania.it (S.N.); francesco.ruotolo@unicampania.it (F.R.); santa.iachini@unicampania.it (T.I.); 2 Laboratory of Clinical Neuropsychology, Neurological Unit of “Ospedali dei Colli”, 80131 Naples, Italy; aleiavarone@gmail.com; 3 Department of Neuroscience, Imaging and Clinical Sciences, “Gabriele d’Annunzio” University of Chieti and Pescara, Via dei Vestini, 33, 66100 Chieti, Italy; mariarosaria.pasciucco@unich.it (M.R.P.); francesca.ferri@unich.it (F.F.); marcello.costantini@unich.it (M.C.); 4 Institute for Advanced Biomedical Technologies, “Gabriele d’Annunzio” University of Chieti and Pescara, Via Polacchi, 11, 66100 Chieti, Italy

**Keywords:** spatial reference frames, interoceptive awareness, health-related quality of life, amnestic Mild Cognitive Impairment, emotional salience

## Abstract

**Background:** Aging involves complex interactions among cognitive, bodily, and spatial processes that may contribute to autonomy and health-related quality of life. Although these processes have been investigated in relation to cognitive functioning, less is known about how spatial and bodily processes are associated with subjective health during cognitive aging. This issue is particularly relevant in amnestic Mild Cognitive Impairment (aMCI), a condition associated with cognitive changes that may affect spatial processing and perception of bodily states. The present study examined associations between egocentric and allocentric spatial processing, assessed through positive, negative, and neutral emotional stimuli, self-reported body awareness, and health-related quality of life in older adults (OA) and individuals with aMCI. **Methods:** Participants completed an egocentric–allocentric spatial task, the Body Perception Questionnaire–Body Awareness (BPQ-BOA), and the Short Form Health Survey (SF-36). Associations were examined separately for the two groups across eight SF-36 domains using bootstrap-based LASSO variable selection followed by multiple regression analyses. **Results:** In OA, Physical Functioning was positively associated with egocentric spatial performance for positive and neutral stimuli, while negative allocentric performance was negatively associated with Emotional Well-Being. In aMCI, Physical Functioning was positively associated with positive allocentric performance and self-reported body awareness, whereas positive egocentric performance and body awareness were negatively associated with Emotional Well-Being and Energy/Fatigue. Emotionally valenced spatial conditions were represented across several models, suggesting that emotional context may be relevant to the observed associations. **Conclusions:** The findings suggest that spatial processing and self-reported body awareness show domain-specific patterns of association with perceived health in OA and aMCI. However, given the cross-sectional design and the small sample size, the distinct patterns observed within each group should be interpreted more as reflecting correlational variations in individual profiles than structural or mechanistic changes in body–environment integration. Further studies using larger samples and longitudinal designs, complemented by behavioral, physiological, and neuroimaging measures, may help establish the reproducibility of these patterns and clarify their underlying processes.

## 1. Introduction

The progressive increase in life expectancy has intensified interest in identifying the mechanisms that support autonomy, social participation, and health-related quality of life (HRQoL) during aging [1,2,3]. According to the World Health Organization framework of Healthy Ageing, well-being depends not only on the absence of disease but also on the preservation of functional abilities that enable individuals to adapt to everyday environments [4,5]. Understanding the cognitive and bodily mechanisms supporting this adaptive capacity is therefore essential for characterizing both healthy and pathological aging.

Within embodied cognition frameworks, adaptive behavior emerges from the continuous interaction between bodily states and environmental representations [6,7]. Two processes are particularly relevant to this interaction: interoception and spatial cognition. Interoception is the perception and interpretation of internal bodily signals, which supports physiological regulation, emotional experience, and self-related processing [8,9,10].

In this study we assessed interoceptive awareness, which is the conscious ability to identify, process and interpret internal body signals [8,9,11]. Interoceptive awareness may play a pivotal role in maintaining health-related well-being and autonomy in older age, as it signals fundamental physiological needs and informs affective and physical appraisals [12]. In this framework, self-reported interoceptive awareness serves as a crucial barometer for daily functioning, shaping how older individuals perceive bodily changes, monitor their physical resources, and sustain overall quality of life [10,13].

In parallel, spatial cognition enables individuals to represent the relationship between their body and the surrounding environment through egocentric (body-centered) and allocentric (environment-centered) reference frames [14,15,16]. These systems rely on partially distinct neural substrates, with egocentric processing primarily supported by fronto-parietal networks and allocentric processing critically dependent on hippocampal integrity [15,17,18].

Healthy aging is characterized by a relative preservation of egocentric representations, whereas allocentric processing shows greater vulnerability, likely reflecting age-related changes affecting medial temporal structures [19,20,21]. This distinction is particularly relevant because spatial cognition contributes not only to navigation but also to mobility, autonomy, and everyday functioning [21,22].

Amnestic Mild Cognitive Impairment (aMCI), representing a possible prodromal stage of Alzheimer’s disease (AD), provides an informative model for investigating specific associations in body–environment integration. Because allocentric representations strongly depend on hippocampal integrity, impairments in allocentric spatial processing are considered among the earliest behavioral markers of AD-related dysfunction [20,23,24,25,26,27,28,29,30].

Beyond spatial cognition, increasing evidence indicates that aging also affects the processing of internal bodily information. Altered interoceptive awareness has been associated with physical functioning and subjective health in older adults [31,32,33,34], while recent studies suggest that its contribution depends on interactions with exteroceptive and multisensory processes rather than on interoception alone [34,35]. Although evidence in aMCI remains limited, neuroimaging findings indicate that early cognitive decline may involve altered functional organization of networks supporting bodily awareness and salience processing [36]. Moreover, emerging evidence suggests that hippocampal systems contribute not only to spatial cognition and episodic memory but also to bodily self-processing [37,38,39,40,41], providing a neurocognitive framework for investigating body–environment integration in aMCI.

Emotional salience represents an additional factor shaping body–environment interactions. Emotional stimuli receive prioritized processing [42,43,44], and recent evidence indicates that emotional valence differentially modulates egocentric and allocentric representations [45]. Although healthy aging is typically characterized by a positivity effect [46,47], emotional processing in MCI appears more heterogeneous [48,49,50], suggesting that emotionally salient stimuli may provide a more ecologically valid framework for investigating body–environment integration.

Although spatial cognition and interoceptive processing have both been linked to aging and cognitive decline, little is known about how they jointly contribute to subjective health or whether their contribution differs between healthy and pathological aging. The present study investigated whether body–environment integration, operationalized through egocentric and allocentric spatial encoding and interoceptive awareness, is associated with different domains of health-related quality of life in older adults and individuals with aMCI. Spatial abilities were assessed using an egocentric–allocentric (Ego-Allo) task [16,21,28,45] involving positive, negative, and neutral emotional stimuli; interoceptive awareness was measured with the Body Perception Questionnaire–Body Awareness scale (BPQ-BOA; [51,52]), and health related quality of life was assessed using the Short Form Health Survey (SF-36; [53,54]).

Based on previous evidence, we hypothesized that interoceptive awareness would be associated with HRQoL, reflecting the theoretically established relevance of interoceptive processing to bodily awareness, subjective health, and well-being. The inclusion of interoceptive awareness was therefore theory-driven and specified a priori. In contrast, given the lack of sufficiently specific evidence to determine a priori which individual egocentric or allocentric spatial measures would be most strongly associated with HRQoL, the selection of spatial predictors was treated as data-driven. We further examined whether the contribution of spatial measures differed between groups, given evidence of age-related vulnerability of allocentric processing and spatial alterations in aMCI. We also examined whether emotionally salient stimuli, particularly positive stimuli, contributed to these associations, reflecting the interplay among spatial cognition, bodily awareness, and emotional processing during healthy and pathological aging.

## 2. Materials and Methods

### 2.1. Participants

Twenty-three participants (12 Older Adults; 11 MCI patients) (M_age_OA_ = 71, SD_age_OA_ = 1.65, M_age_MCI_ = 73, SD_age_MCI_ = 4.75, M_education_OA_ = 13.66, SD_education_OA_ = 2.01, M_education_MCI_ = 12, SD_education_MCI_ = 5) were recruited. Informed consent was obtained from all participants. The aMCI patients’ mean MMSE score was 25.18, SD = 3.43 (corrected = 26.53, SD = 3.78), and the MoCA score was 20.09, SD = 4.41 (corrected = 25.62, SD = 2.88). The OA group had a mean score at an MMSE of 29.25, SD = 1.14 (corrected = 27.65, SD = 2.30), and the MoCA score was 24.17, SD = 2.55 (corrected = 23.55, SD = 2.61). Older participants were recruited at the Laboratory of Cognitive Science and Immersive Virtual Reality, in conformity with the requirements of the local Institutional Ethics Committee [N. 36/2023] and the 2013 Declaration of Helsinki. Patients were recruited at the Ospedale dei Colli (Ospedale dei Colli Aminei, C.T.O., Naples, Italy) in accordance with the hospital’s ethics committee requirements [AOC-0029533-2024].

### 2.2. Experimental Sessions

The study consisted of two sessions. In the first session, participants completed two neuropsychological tests (MMSE, MoCA) [55,56,57,58] and questionnaires on interoceptive awareness (BPQ) and daily living functioning (SF-36) [53,54]. In the second session, participants completed a modified version of the Ego/Allo Task [16,21,28,45] with objects. The entire experiment was conducted in a quiet and comfortable room.

#### 2.2.1. Neuropsychological Assessment

All participants with aMCI underwent a comprehensive neuropsychological and clinical assessment in a hospital setting. Diagnostic criteria for aMCI were based on international guidelines [59,60,61] requiring: (i) presence of cognitive complaints; (ii) objective episodic memory impairment, defined as performance at least 1.5 SD below age- and education-adjusted normative means (or Equivalent Score = 0) on standardized episodic memory tasks; (iii) preserved functional autonomy in activities of daily living; and (iv) absence of dementia. The assessment battery included measures of global cognitive functioning (MMSE [55,56] and MoCA [57,58]), verbal and visuospatial episodic memory (Rey 15-Word Test [62] immediate and delayed recall; Rey–Osterrieth Complex Figure Test, copy and delayed recall [63]; Free and Cued Selective Reminding Test [FCSRT] [64]), working memory (Digit Span Forward and Backward) [65], attention and executive functioning (Trail Making Test A and B; Stroop Test) [66,67], verbal fluency (phonemic and semantic fluency) [62,68], functional autonomy (ADL and IADL) [69,70] and affective symptoms (Beck Depression Inventory and Hamilton rating scales) [71,72]. Following the completion of the neuropsychological and clinical assessment, the diagnosis of aMCI was formulated by a neurologist and a clinical neuropsychologist. Exclusion criteria for the clinical group included a history of major stroke or focal brain lesions, traumatic brain injury, neurological conditions other than primary cognitive decline, and major psychiatric disorders.

The older adult (OA) cohort exhibited no history of neurologic, psychiatric, or systemic medical conditions potentially affecting cognitive performance (e.g., Alzheimer’s disease, multiple sclerosis, or Parkinson’s disease). The Mini Mental State Examination (MMSE) [55,56] and Montreal Cognitive Assessment (MoCA) [57,58] were administered to all participants. The MMSE and MoCA scores were calculated according to standard scoring procedures. Corrected scores were obtained according to the normative adjustments provided by Measso et al. [56] for the MMSE and Santangelo et al. [58] for the MoCA. According to these normative references, the cut-off scores considered were 23.80 for the MMSE and 15.5 for the MoCA. Both raw and corrected scores were carefully re-checked to ensure the accuracy of the calculations.

Additionally, emotional processing was assessed via the 20-item Toronto Alexithymia Scale (TAS-20) [73], a well-established self-report tool that evaluates difficulties in identifying and describing emotions. Participants responded using a 5-point Likert scale (1 = strongly disagree; 5 = strongly agree) across three main subscales: difficulty identifying feelings, difficulty describing feelings, and externally oriented thinking. Total TAS-20 scores range from 20 to 100, categorized into non-alexithymic (≤51), borderline (52–60), or clinically significant alexithymia (≥61). The scale possesses robust psychometric properties, with a good internal consistency (Cronbach’s alpha = 0.81) and test–retest reliability (0.77, *p* < 0.01). The TAS-20 was administered exclusively as a screening instrument to identify and exclude potential participants with clinically significant alexithymia in both groups; however, no participants were excluded on this basis, as all obtained scores were within the non-alexithymic range (≤51).

#### 2.2.2. Interoceptive Awareness and Daily Living Functioning

To assess interoceptive awareness, participants completed the Body Perception Questionnaire (BPQ; [51,52]), a well-established self-report instrument evaluating subjective awareness of bodily signals and autonomic reactivity. Specifically, we administered the validated 22-item Italian Short Form [BPQ-22; 51], which is organized into a three-factor structure encompassing body awareness (BOA), supradiaphragmatic reactivity (SUP), and subdiaphragmatic reactivity/body awareness (BOA/SUB). In the present study, analyses focused on the Body Awareness subscale (BPQ-BOA), which targets subjective awareness of bodily sensations primarily associated with the upper body and physiological state monitoring. Recent literature suggests that self-reported interoceptive awareness measured by the BPQ may tap into dimensions of heightened somatic focus, hypervigilance, or somatization rather than purely neutral or adaptive bodily monitoring [74,75].

To assess daily living functioning, the SF-36 was administered [53,54]. This 36-item self-report questionnaire evaluates perceived health status across eight distinct dimensions encompassing functional status, well-being, and global health evaluations [76]. The comprehensive tool measures Physical Functioning, Role Limitations Due to Physical Health, Role Limitations Due to Personal or Emotional Problems, Energy/Fatigue, Emotional Well-Being, Social Functioning, Bodily Pain, and General Health. Each subscale can be analyzed independently, with scores ranging from 0 to 100 (where lower scores indicate poorer perceived health and higher scores reflect better health status).

#### 2.2.3. Ego Allo Task

##### Setting and Stimuli

The study was carried out in a soundproof room at the Cognitive Science and Immersive Virtual Reality Laboratory (CS-IVR, Department of Psychology, University of Campania “Luigi Vanvitelli”, Italy). Virtual environments were rendered using Unity software version 2022.3.16f1 [77] and administered via Oculus Quest (Meta Platforms Inc., Menlo Park, CA, USA) The triads were arranged according to the following criteria: (i) the distances between the objects were clearly perceivable; (ii) metric distances were established such that the degree of metrical difficulty was identical for allocentric (“allo”) and egocentric (“ego”) judgments; (iii) each triad was positioned relative to a participant’s midsagittal plane, providing a stable egocentric reference point. To ensure that all triads were presented in the same way, we adopted the same spatial distance configuration used in previous studies [16,21,28,45,78,79,80]. Metric distances were established to ensure the same level of difficulty for egocentric and allocentric judgments; for example, the central object (T) served as the allocentric reference stimulus. The allocentric distances were as follows: object “a”—object “b” = 11 cm, object “c”—object “b” = 18 cm. As for the egocentric distances, object “a” and object “c” were respectively 19 cm and 12 cm from the participant’s median sagittal plane. The metric difference between the two stimuli closest to the participant (19–12 cm) and between objects (18–11) was identical, namely 7 cm. The virtual room maintained constant dimensions across trials, while the distances between objects preserved the same gradient of metric difficulty used in the original paradigm. Stimuli consisted of 36 virtual objects organized into distinct triads. Objects were modelled and measured in SketchUp (v2023; Trimble Inc., Westminster, CO, USA) [81], optimized in Blender (v4.2; Blender Foundation, Amsterdam, The Netherlands) [82], and imported into Unity (v2022.3.16f1; Unity Technologies, San Francisco, CA, USA). Stimuli were displayed within a 10 m × 7 m virtual room across separate experimental blocks. Stimuli were categorized by emotional valence (positive, neutral, negative). Representative object triads included: positive (ball, teddy bear, toy train), neutral (sphere, cone, pyramid), and negative (bomb, dynamite, gun). We used a modified version of Ego Allo Task [16] with objects. We manipulated emotional valence (positive, neutral and negative) and frame of reference (egocentric or allocentric). Participants were asked to provide egocentric judgments (e.g., “Is X closest to you?”) or allocentric judgments (e.g., “Is X closest to Y?”). To validate the emotional valence of the stimuli, a questionnaire was created using Google Forms (Google LLC, Mountain View, CA, USA; https://www.google.com/forms/about/, accessed on 10 May 2026) and administered to an independent sample of 28 participants, including 13 younger adults and 15 older adults. Participants were asked to observe each object and categorize the emotion or valence conveyed by selecting one of four options (positive, negative, neutral, or other). Subsequently, they rated the emotional intensity of the objects (positivity, negativity, and neutrality) using 7-point Likert scales. They also evaluated the perceived valence (i.e., how unhappy or happy they felt while viewing the image) and arousal (i.e., how calm or stimulated they felt while viewing the image). Finally, participants rated the perceived aesthetic pleasantness of the object and its perceived familiarity, defined as how familiar the object appeared regardless of whether the participant had actually seen it before. All stimuli, including the neutral geometric stimuli, were evaluated during the validation procedure. Results of validation showed that for older adults, positive and negative stimuli showed comparable arousal levels, whereas neutral stimuli were characterized by lower arousal (positive: M = 5.58, SD = 0.17; negative: M = 5.86, SD = 0.43; neutral: M = 3.39, SD = 0.27). Specifically, for each selected stimulus, the intended valence was identified as the most frequently endorsed category by at least 75% of the validators. Valence ratings followed the expected pattern, with positive, neutral, and negative stimuli receiving progressively lower ratings (positive: M = 5.21, SD = 0.71; neutral: M = 3.53, SD = 0.45; negative: M = 2.69, SD = 0.60). Recognition and familiarity were generally high across categories (recognition: positive M = 6.28, SD = 0.20; neutral M = 5.98, SD = 0.34; negative M = 5.88, SD = 0.45; familiarity: positive M = 6.19, SD = 1.05; neutral M = 5.70, SD = 0.31; negative M = 5.21, SD = 0.65). Emotional categorization was consistent with the intended categories (positive: M = 5.49, SD = 0.56; neutral: M = 5.79, SD = 0.34; negative: M = 5.51, SD = 0.54). A similar pattern was observed in younger adults, with comparable arousal levels for positive and negative stimuli and lower arousal for neutral stimuli (positive: M = 5.16, SD = 0.59; negative: M = 5.68, SD = 0.80; neutral: M = 2.91, SD = 0.29). Valence ratings again followed the expected pattern (positive: M = 5.38, SD = 0.76; neutral: M = 3.54, SD = 0.21; negative: M = 2.43, SD = 0.69). Recognition and familiarity were generally high across categories (recognition: positive M = 6.28, SD = 0.39; neutral M = 5.17, SD = 0.34; negative M = 5.87, SD = 0.43; familiarity: positive M = 6.19, SD = 0.35; neutral M = 4.87, SD = 0.33; negative M = 5.26, SD = 0.64). Emotional categorization was consistent with the intended categories (positive: M = 5.67, SD = 0.48; neutral: M = 5.05, SD = 0.24; negative: M = 5.37, SD = 0.64).

### 2.3. Procedure and Experimental Design

After receiving all the necessary information regarding the task, participants were asked to wear the VR headset. Prior to commencing the experimental task, a training session was administered to allow participants to familiarize themselves with the virtual environment and fully comprehend the procedure. During this phase, participants observed stimuli appearing within the virtual room and were required to memorize both the distance of the stimuli relative to themselves (egocentric judgment) and the distance between the stimuli (allocentric judgment). Two types of questions were presented: “Which stimulus is closest/farthest to/from you?” (egocentric judgment); “Which stimulus is closest/farthest to/from another stimulus?” (allocentric judgment). To respond, participants pressed “L” for “YES” and “K” for “NO”. Once the training session was completed and all instructions were verified as clear, participants proceeded to the experimental task, which was identical to the training phase.

#### Testing Phase and Experimental Design

The experimental session comprised three distinct blocks (positive, neutral, and negative), presented in a quasi-randomized order. The task included a total of 24 trials, divided evenly between spatial reference frame and emotional valence: 12 egocentric trials (4 positive, 4 neutral, and 4 negative) and 12 allocentric trials (4 positive, 4 neutral, and 4 negative). For each trial, response accuracy was recorded dichotomously as 0 (incorrect) or 1 (correct). For each participant, six valence-specific spatial indices, Positive Egocentric (PE), Neutral Egocentric (NTE), Negative Egocentric (NE), Positive Allocentric (PA), Neutral Allocentric (NTA), and Negative Allocentric (NA), were computed as the mean accuracy across the four corresponding trials. The overall immersive virtual reality experiment lasted approximately 30 min.

### 2.4. Data Analysis

All statistical analyses were performed using R (v4.3.2; R Core Team, Vienna, Austria) [83]. To examine the associations between interoception, spatial predictors, and SF-36 subscale scores (Physical Functioning, Social Functioning, Role Limitations Due to Physical Health, Role Limitations Due to Emotional Health, Emotional Well-Being, Pain, Energy/Fatigue, and General Health), analyses were conducted separately in the aMCI and older-adult groups. The complete analytical procedure was therefore repeated for each of the eight SF-36 subscales, which were treated as continuous outcomes. For each outcome, BOA and six spatial indices (NE, NA, NTE, NTA, PE, and PA) were entered as candidate predictors.

A bootstrap-based LASSO (Least Absolute Shrinkage and Selection Operator) variable-selection procedure was applied to each outcome. LASSO performs coefficient shrinkage and variable selection by constraining the absolute magnitude of regression coefficients, with some coefficients potentially reduced to zero [84]. For each group and SF-36 subscale, 1000 bootstrap samples were generated with replacement. LASSO models were fitted to each bootstrap sample, with the regularization parameter (λ) selected by five-fold cross-validation using the value minimizing the cross-validated mean squared error (λmin). Predictors with non-zero coefficients at λmin were considered selected, and selection frequency was calculated for each predictor as the percentage of bootstrap samples in which it was selected. This resampling-based approach follows previous applications of bootstrap-enhanced penalized regression for assessing variable-selection stability [85] and is related to the broader framework of stability selection [86].

A selection frequency of ≥50% was used as an exploratory criterion for retaining spatial predictors. BOA was retained regardless of its selection frequency because it was the prespecified predictor of theoretical interest. The resulting predictors were entered into a standard multiple linear regression model for each SF-36 subscale. Model fit was assessed using R^2^, adjusted R^2^, the overall F-test, regression coefficients, standard errors, and *p*-values.

The predictive performance of each final model was evaluated using leave-one-out cross-validation (LOOCV). At each iteration, the model was refitted after leaving out one participant, and the outcome for the omitted participant was predicted. Predictor selection was repeated within each training fold, with the left-out participant excluded from both the selection and model-fitting procedures, thereby preventing information leakage. Performance was quantified using the root mean squared error (RMSE), mean absolute error (MAE), and predictive R^2^. Thus, the complete analytical procedure was performed eight times, once for each SF-36 subscale, separately within the aMCI and older-adult groups. Results from all eight analyses are reported, with the principal findings presented in the main text and the complete results, including non-significant findings, provided in the Supplementary Materials. As a complementary exploratory analysis, permutation tests based on five thousand random permutations of the outcome were used to assess whether the observed overall model fit was greater than expected under random correspondence between predictors and outcome [87,88].

## 3. Results

For Physical Functioning (PF), predictor selection frequencies differed between groups. In the aMCI group, NTA had the highest selection frequency (69.1%), followed by PA (52.0%) and PE (50.6%). In older adults, PE was most frequently selected (72.4%), followed by NTE (63.0%). The remaining spatial predictors were selected in fewer than 50% of bootstrap samples in both groups. Based on the prespecified exploratory criterion of ≥50% selection frequency, the final models therefore included BOA, NTA, PA, and PE in the aMCI group, and BOA, PE, and NTE in the older-adult group.

In the aMCI group, the multiple regression analysis with Physical Functioning (PF; SF-36) as the criterion variable yielded a significant model, F(4, 6) = 4.521, *p* = 0.05, R^2^ = 0.751, adjusted R^2^ = 0.585. BPQ-BOA and Positive Allo emerged as significant predictors of perceived physical functioning (Table 1a). Specifically, higher self-reported body awareness and higher allocentric spatial performance for positive stimuli were associated with better perceived physical functioning. Neutral Allo and Positive Ego also showed positive associations with Physical Functioning, although these did not reach conventional statistical significance. Permutation testing indicated that the observed model fit was unlikely to be explained by chance (R^2^ = 0.750, *p* = 0.05).

In the older-adult (OA) group, the multiple regression analysis also yielded a significant model, F(3, 8) = 9.342, *p* = 0.005, R^2^ = 0.778, adjusted R^2^ = 0.695. Positive Ego and Neutral Ego significantly predicted Physical Functioning (Table 1b). Specifically, higher egocentric spatial performance for positive and neutral stimuli was associated with better perceived physical functioning, whereas BPQ-BOA was not significantly associated with Physical Functioning in this group. Permutation testing indicated that the observed model fit was unlikely to be explained by chance (R^2^ = 0.778, *p* = 0.009).

For Emotional Well-Being (EWB), predictor selection frequencies differed between groups. In the aMCI group, NTE had the highest selection frequency (54.5%), followed by PE (54.0%). In older adults, PA had the highest selection frequency (59.1%), followed by NA (57.3%) and PE (51.5%). The remaining spatial predictors were selected in fewer than 50% of bootstrap samples in both groups. Based on the prespecified exploratory criterion of ≥50% selection frequency, the final models therefore included BOA, NTE, and PE in the aMCI group, and BOA, PA, NA, and PE in the older-adult group.

In the aMCI group, the multiple regression analysis with Emotional Well-Being (BE; SF-36) as the criterion variable yielded a significant model, F(3, 7) = 5.149, *p* = 0.034, R^2^ = 0.688, adjusted R^2^ = 0.555. BPQ-BOA and Positive Ego emerged as significant predictors of Emotional Well-Being (Table 1c). Specifically, higher self-reported body awareness was associated with lower perceived emotional well-being, while higher spatial performance in egocentric judgments for positive stimuli was also associated with lower perceived emotional well-being. NTE showed a positive association with Emotional Well-Being, although this did not reach conventional statistical significance. Finally, permutation testing indicated that the observed model fit was unlikely to be explained by chance (R^2^ = 0.688, *p* = 0.03).

In the older-adult (OA) group, the multiple regression analysis also yielded a significant model, F(4, 7) = 5.035, *p* = 0.031, R^2^ = 0.742, adjusted R^2^ = 0.595. NA emerged as a significant predictor of Emotional Well-Being (Table 1d). Specifically, higher negative allocentric spatial performance was associated with lower perceived emotional well-being. BPQ-BOA also showed a negative association with Emotional Well-Being, although this did not reach conventional statistical significance, whereas PA and PE were not significantly associated with the outcome. Finally, permutation testing indicated that the observed model fit was unlikely to be explained by chance (R^2^ = 0.742, *p* = 0.03).

LOOCV provided an out-of-sample assessment of these models. For aMCI, the model yielded an RMSE of 26.94, an MAE of 22.12, and a predictive R^2^ of 0.23, indicating that the model retained positive predictive explanatory value when evaluated on participants not used to fit the corresponding model. In older adults, the RMSE was 18.19, the MAE was 13.52, and the predictive R^2^ was 0.029. For OA, the model yielded an RMSE of 21.53, an MAE of 16.30, and a predictive R^2^ of 0.047. In older adults, the RMSE was 20.437, the MAE was 14.929, and the predictive R^2^ was −1.080.

For Energy/Fatigue (EF), predictor selection frequencies differed between groups. In the aMCI group, PE had the highest selection frequency (68.5%), followed by NTE (52.4%). In older adults, PE was the only spatial predictor selected in more than 50% of bootstrap samples (50.5%). The remaining spatial predictors were selected in fewer than 50% of bootstrap samples in both groups. Based on the prespecified exploratory criterion of ≥50% selection frequency, the final models therefore included BOA, PE, and NTE in the aMCI group, and BOA and PE in the older-adult group.

In the aMCI group, the multiple regression analysis with Energy/Fatigue (EF; SF-36) as the criterion variable yielded a significant model, F(3, 7) = 6.917, *p* = 0.017, R^2^ = 0.748, adjusted R^2^ = 0.640. BPQ-BOA and Positive Ego emerged as significant predictors of Energy/Fatigue (Table 1e). Specifically, higher self-reported body awareness and higher spatial performance in egocentric judgments for positive stimuli were associated with lower perceived energy/fatigue scores. NTE showed a positive association with Energy/Fatigue, although this did not reach conventional statistical significance. Finally, permutation testing indicated that the observed model fit was unlikely to be explained by chance (R^2^ = 0.747, *p* = 0.01).

In the older-adult (OA) group, the multiple regression analysis with Energy/Fatigue as the criterion variable did not yield a significant model, F(2, 9) = 0.848, *p* = 0.460, R^2^ = 0.159, adjusted R^2^ = −0.028. Neither BPQ-BOA nor Positive Ego significantly predicted Energy/Fatigue in this group (Table 1f).

LOOCV provided an out-of-sample assessment of these models. For aMCI, the model yielded an RMSE of 15.75, an MAE of 12.97, and a predictive R^2^ of 0.378, indicating positive predictive explanatory value when evaluated on participants not used to fit the corresponding model. In older adults, the RMSE was 20.43, the MAE was 15.09, and the predictive R^2^ was −2.404. Finally, permutation testing indicated that the observed model fit was unlikely to be explained by chance (R^2^ = 0.158, *p* = 0.457).

## 4. Discussion

The present study investigated the associations between spatial reference frame encoding, assessed through egocentric and allocentric judgments involving emotional stimuli, self-reported body awareness, and different domains of health-related quality of life in cognitively older adults (OA) and individuals with amnestic Mild Cognitive Impairment (aMCI).

Overall, the results are consistent with the idea that subjective perception of health is related to the interaction between spatial cognition, body awareness, and emotional processing, rather than to cognitive or bodily functions considered in isolation. The exploratory analyses revealed distinct patterns of associations within each group. At this stage, the associations between spatial processing, self-reported body awareness, and perceived health should be interpreted more as reflecting correlational variations in individual profiles than structural or mechanistic changes in body–environment integration.

This interpretation is broadly compatible with the embodied cognition framework, which emphasizes the interaction between bodily states and environmental representations in adaptive behavior [6,7]. Previous evidence has also suggested that interoceptive processes may contribute to successful aging through their interaction with exteroceptive and multisensory representations supporting functional abilities and subjective health [31,32]. The present findings, although preliminary, are in line with this perspective by suggesting that the association between spatial and bodily processes and perceived health may not be uniform across different stages of cognitive aging.

In older adults, perceived physical functioning was associated with egocentric spatial performance for positive and neutral stimuli. Higher performance in these egocentric conditions was related to better perceived physical functioning, whereas self-reported body awareness was not significantly associated with this domain. This pattern is compatible with evidence suggesting that egocentric representations may be relatively preserved in healthy aging compared with allocentric representations, which can be more sensitive to age-related changes [16,17,18]. Because egocentric representations support body-centered interactions, action planning, and navigation in familiar environments [15], their association with perceived physical functioning may reflect the relevance of body-centered spatial processing for everyday functioning and subjective physical competence.

In aMCI, perceived physical functioning showed a different pattern of associations. Both self-reported body awareness and allocentric spatial performance for positive stimuli were positively associated with Physical Functioning, while Neutral Allo and Positive Ego showed positive but non-significant associations. The involvement of allocentric processing is of potential interest because allocentric representations are strongly associated with medial temporal and hippocampal systems and may be particularly sensitive to cognitive changes occurring in the early stages of Alzheimer’s disease [15,17,23,27]. However, given the exploratory nature of the present findings, this suggestion deserves further studies.

The different patterns observed in OA and aMCI may be compatible with the possibility that spatial and bodily processes are differently associated across stages of cognitive aging. Drawing inspiration from the literature, we might say that the coexistence of allocentric processing and body awareness in the aMCI model may be consistent with the broader hypothesis that body–environment integration can assume different configurations in the presence of cognitive changes. However, differences in significant predictors within separate within-group regressions cannot be interpreted as evidence of between-group differences. Hence, these observations should be interpreted with caution and regarded as descriptive and exploratory. These findings provide an initial basis for considering possible differences in the coordination of spatial and bodily processes, rather than demonstrating a specific reorganization of these mechanisms.

The association between body awareness and Physical Functioning in aMCI may also be relevant to the interpretation of subjective health. Greater self-reported awareness of bodily sensations was associated with better perceived physical functioning in this group. Given that the BPQ-BOA assesses self-reported body awareness rather than interoceptive accuracy, this finding may reflect differences in subjective attention to bodily states or in the way physical sensations are incorporated into health-related self-evaluations. Self-reported body awareness may also capture aspects of somatic focus or heightened attention to bodily sensations [74,75]. Complementary behavioral and physiological measures may help clarify the nature of this association in future research.

A different pattern emerged for Emotional Well-Being in aMCI. The overall model was associated with Emotional Well-Being, with higher body awareness and higher Positive Ego performance being associated with lower perceived emotional well-being. Neutral Ego showed a positive, although non-significant, association. These findings should not be interpreted as indicating that better spatial performance or greater body awareness has a detrimental effect on emotional well-being. Rather, they may reflect individual differences in the way cognitive and bodily information is monitored and incorporated into subjective evaluations of well-being. In particular, greater awareness of bodily sensations or better performance on certain spatial tasks may coexist with greater awareness of subtle changes in cognitive or physical functioning, potentially influencing self-reported emotional health. This possibility is compatible with previous work documenting variability in awareness and insight in individuals with MCI [89,90], but remains a hypothesis that requires further investigation.

A related pattern was observed for Energy/Fatigue in aMCI. Both Positive Ego performance and body awareness were negatively associated with Energy/Fatigue, whereas Neutral Ego showed a positive but non-significant association. Given the scoring of the SF-36 domain, these findings indicate that higher egocentric performance for positive stimuli and greater self-reported body awareness were associated with lower perceived energy and greater fatigue. One possible interpretation is that individuals who are more attentive to bodily states and who show better performance in some spatial conditions may also report bodily sensations and fatigue more prominently. Alternative explanations, including differences in symptom monitoring or response tendencies, may also account for this pattern. Thus, the present finding may be viewed as an observation that can inform future hypotheses regarding the relationship between spatial and bodily processing and subjective fatigue.

Together, the findings across Physical Functioning, Emotional Well-Being, and Energy/Fatigue in aMCI showed both convergent and domain-specific patterns. Body awareness was associated with all three domains, whereas Positive Ego performance was associated with both Emotional Well-Being and Energy/Fatigue. In contrast, Positive Allo performance was specifically associated with Physical Functioning. Thus, while body awareness and egocentric processing showed some convergence across subjective health domains, allocentric processing appeared more specifically related to perceived physical functioning. This pattern may be compatible with the possibility that different aspects of subjective health draw on partially distinct combinations of spatial and bodily processes.

In the analysis, emotionally salient conditions were frequently represented among the selected predictors. This observation raises the possibility that emotional context may contribute to the associations between spatial processing, body awareness, and subjective health. Emotional stimuli engage neural systems involved in memory, emotion, and salience detection [42,43,44], and previous evidence indicates that emotional valence can differentially influence egocentric and allocentric spatial representations [45].

The frequent involvement of positive stimuli in the observed associations may also be considered in relation to the “positivity effect” described in healthy aging [46,47]. In aMCI, the involvement of both positive and neutral conditions across models suggests that the contribution of emotional context to the associations between spatial processing and subjective health may vary according to cognitive status and the specific health domain considered.

## 5. Limitations

Several limitations must be considered. First, the sample size was relatively small, particularly in the aMCI group (*n* = 11), which may have limited the detection of small-to-moderate associations and affected the precision and generalizability of the estimates. Second, the spatial indices were derived from a limited number of trials per emotional condition. Although the stimuli were validated in independent samples of healthy younger and older adults and correctly recognized by all participants, valence, arousal, and familiarity were not specifically assessed in the aMCI group, and differences in stimulus appraisal or emotional processing may therefore be relevant to the observed patterns. Third, given the number of variables assessed, the problem of multiplicity remains substantial; therefore, the pattern of results should be interpreted as hypothesis-generating rather than confirmatory. Fourth, the cross-sectional design provides information on associations but does not allow temporal or causal relationships to be established. The observed patterns may also reflect individual differences in symptom monitoring, bodily focus, or response tendencies. Relatedly, differences in the pattern of significant predictors across groups do not, in themselves, establish distinct underlying mechanisms, and these group-level variations should be viewed as descriptive rather than demonstrative of distinct neurocognitive profiles. Comparisons between OA and aMCI should further be considered in light of differences in age and cognitive status, which cannot be fully separated from group membership in the present design. In addition, the BPQ-BOA assesses self-reported body awareness rather than objective interoceptive accuracy or sensitivity.

Finally, the exploratory analyses included several spatial measures, emotional conditions, and SF-36 domains. The associations observed, including the relatively high explained variance estimates in some models, should therefore be regarded as initial findings. Further research with larger samples and longitudinal designs, complemented by behavioral, physiological, and neuroimaging measures, will help clarify the reproducibility and broader significance of these relationships.

## 6. Conclusions

Overall, the present findings suggest that spatial processing and self-reported body awareness are associated with different domains of health-related quality of life, with partially distinct patterns observed in OA and individuals with aMCI. In OA, Physical Functioning was primarily associated with egocentric spatial performance, whereas in aMCI it was associated with Positive Allo performance and body awareness; in addition, Positive Ego performance and body awareness were also associated with Emotional Well-Being and Energy/Fatigue.

These findings suggest that the relationships between spatial processing, bodily awareness, and subjective health may vary across stages of cognitive aging and across health domains. In theory, the observed within-group associations could be compatible with different configurations of the body–environment relationship and with changes in how spatial and bodily processes are coordinated. The involvement of allocentric processing may also be compatible with hypotheses concerning the contribution of hippocampal or medial temporal systems, although the present study provides no direct evidence for the involvement of these neural systems. However, given the cross-sectional design, small sample size and self-report measures, these observations should be interpreted with caution. Future longitudinal investigations with larger samples are required to test formal interactions and determine the generalizability of these exploratory linkages.

## Figures and Tables

**Table 1:** (a) The table shows the effect of predictors that contributed significantly to the model in relation to Physical Functioning as a criterion in the aMCI group. (b) The table shows the effect of predictors that contributed significantly to the model in relation to Physical Functioning as a criterion in the Older Adults group. (c) The table shows the effect of predictors that contributed significantly to the model in relation to Emotional Well-Being as a criterion in the aMCI group. (d) The table shows the effect of predictors that contributed significantly to the model in relation to Emotional Well-Being as a criterion in the Older Adults group. (e) The table shows the effect of predictors that contributed significantly to the model in relation to Energy/Fatigue as a criterion in the aMCI group. (f) The table shows the effect of predictors that contributed significantly to the model in relation to Energy/Fatigue as a criterion in the Older Adults group.

Variable	Estimate	Std.Error	CI_Lower	CI_Upper	t.Value	*p*
(a)						
(Intercept)	−291,571	107,904	−555,60	−27,54	−2702	0.035
BOA	10,797	3673	1810	19,783	2940	0.026
NTA	0.482	0.235	−0.094	1058	2048	0.086
PA	0.986	0.370	0.080	1892	2664	0.037
PE	1523	0.641	0.046	3091	2376	0.055
(b)						
(Intercept)	31,556	21,178	−17,280	80,392	1490	0.175
BOA	−2247	1438	−5564	1070	−1562	0.157
PE	0.549	0.119	0.275	0.823	4626	0.002
NTE	0.435	0.181	0.018	0.853	2404	0.043
(c)						
(Intercept)	241,781	66,368	84,845	398,717	3643	0.008
BOA	−5825	2139	−10,884	−0.767	−2723	0.030
NTE	0.274	0.195	−0.188	0.736	1403	0.203
PE	−1377	0.454	−2451	−0.303	−3033	0.019
(d)						
(Intercept)	151,539	23,318	96,400	206,677	6499	0.000
BOA	−3298	1435	−6690	0.094	−2299	0.055
PA	−0.150	0.112	−0.414	0.114	−1345	0.220
NA	−0.489	0.141	−0.822	−0.156	−3475	0.010
PE	−0.016	0.110	−0.275	0.243	−0.147	0.887
(e)						
(Intercept)	252,648	54,056	124,826	380,471	4674	0.002
BOA	−4865	1742	−8985	−0.745	−2792	0.027
PE	−1512	0.370	−2387	−0.638	−4089	0.005
NTE	0.082	0.159	−0.294	0.458	0.516	0.622
(f)						
(Intercept)	68,393	21,436	19,902	116,885	3191	0.011
BOA	−1248	1491	−4621	2125	−0.837	0.424
PE	0.134	0.131	−0.162	0.429	1023	0.333

## Data Availability

The data presented in this study are available on request from the corresponding author. The data are not publicly available due to privacy and ethical restrictions.

## Supplementary Materials

The following supporting information can be downloaded at: https://www.mdpi.com/article/10.3390/jcm15207787/s1, File S1: Complete LASSO selection frequencies and multiple linear regression models for all eight SF-36 subscales in older adults (OA) and individuals with amnestic Mild Cognitive Impairment (aMCI).

## Abbreviations

The following abbreviations are used in this manuscript:

HRQoL	Health-related quality of life
aMCI	Amnestic mild cognitive impairment
MCI	Mild cognitive impairment
OA	Older adults
PF	Physical Functioning
EW	Emotional Well Being
EF	Energy Fatigue
