# Supplementary material for: When Body and Space Shape Perceived Health: Spatial and Interoceptive Processes in Amnestic Mild Cognitive Impairment
Source: J Clin Med. 2026 Oct 9;15(20):7787. doi: 10.3390/jcm15207787 (PMC13705349; doi:10.3390/jcm15207787)
Supplement: Supplementary file 1 [file jcm-15-07787-s001.zip › jcm-4505498-supplementary.pdf]

Supplementary Materials

File S1. Complete LASSO selection frequencies and multiple linear regression models for all eight SF-36 subscales in older adults (OA) and individuals with amnestic Mild Cognitive Impairment (aMCI)

Physical Functioning (**PF**)

Predictor selection based on bootstrap-enhanced LASSO procedure. Predictor>50%.

Predictor	aMCI_percent	Healthy_percent	Difference
NTA	69,1	44,2	24,9
PE	50,6	72,4	-21,8
NTE	42,8	63	-20,2
NE	30	38	-8
PA	52	44,1	7,9
NA	40,1	38,2	1,9

Multiple linear regression models for each group.

Group	N	Formula	R2	Adjusted_R2	F	df1	df2	Model_p
aMCI	11	FU ~ BOA + NTA + PA + PE	0,751	0,585	4,521	4	6	0,050
Healthy	12	FU ~ BOA + PE + NTE	0,778	0,695	9,342	3	8	0,005

*The degrees of freedom are: df1 = selected predictors; df2 = N-k-1 (N = sample size; K = selected predictors; 1 = intercept).

The table shows the effect of predictors that contributed significantly to the model in relation to PH as a criterion in the aMCI group.

Variable	Estimate	Std..Error	CI_lower	CI_upper	t.value	*p*
(Intercept)	-291,571	107,904	-586,17	94,35	-2,702	0,035
BOA	10,797	3,673	-4,17	20,34	2,940	0,026
NTA	0,482	0,235	-0,18	1,47	2,048	0,086
PA	0,986	0,370	-0,32	2,24	2,664	0,037
PE	1,523	0,641	-0,50	3,14	2,376	0,055

The table shows the effect of predictors that contributed significantly to the model in relation to PH as a criterion in the Healthy group.

Variable	Estimate	Std..Error	CI_lower	CI_upper	t.value	*p*
(Intercept)	31,556	21,178	-15,170	131,720	1,490	0,175
BOA	-2,247	1,438	-7,571	1,002	-1,562	0,157
PE	0,549	0,119	-0,136	0,798	4,626	0,002
NTE	0,435	0,181	0,098	0,865	2,404	0,043

Emotional Well-Being (**EWB**)

Predictor selection based on bootstrap-enhanced LASSO procedure. Predictor>50%.

Predictor	aMCI_percent	Healthy_percent	Difference
NA	26,3	57,3	-31
NTE	54,5	28,2	26,3
NE	35,8	46,8	-11
PA	49,7	59,1	-9,4
NTA	46,2	38,8	7,4
PE	54	51,5	2,5

Multiple linear regression models for each group.

Group	N	Formula	R2	Adjusted_R2	F	df1	df2	Model_p
aMCI	11	BE ~ BOA + NTE + PE	0,688	0,555	5,149	3	7	0,034
Healthy	12	BE ~ BOA + PA + NA_pred + PE	0,742	0,595	5,035	4	7	0,031

*The degrees of freedom are: df1 = selected predictors; df2 = N-k-1 (N = sample size; K = selected predictors; 1 = intercept).

The table shows the effect of predictors that contributed significantly to the model in relation to EWB as a criterion in the aMCI group.

Variable	Estimate	Std..Error	CI_lower	CI_upper	t.value	*p*
(Intercept)	241,781	66,368	70,080	455,168	3,643	0,008
BOA	-5,825	2,139	-13,601	-0,012	-2,723	0,030
NTE	0,274	0,195	-0,615	0,709	1,403	0,203
PE	-1,377	0,454	-2,757	-0,147	-3,033	0,019

The table shows the effect of predictors that contributed significantly to the model in relation to EWB as a criterion in the Healthy group.

Variable	Estimate	Std..Error	CI_lower	CI_upper	t.value	*p*
(Intercept)	151,539	23,318	87,874	231,192	6,499	0,000
BOA	-3,298	1,435	-8,105	0,153	-2,299	0,055
PA	-0,150	0,112	-0,496	0,208	-1,345	0,220
NA	-0,489	0,141	-0,903	0,000	-3,475	0,010
PE	-0,016	0,110	-0,935	0,187	-0,147	0,887

Energy/Fatigue (**EF**)

Predictor selection based on bootstrap-enhanced LASSO procedure. Predictor>50%.

Predictor	aMCI_percent	Healthy_percent	Difference
NTE	52,4	31,8	20,6
PE	68,5	50,5	18
NTA	36,7	28,5	8,2
PA	36,3	28,1	8,2
NE	36,2	30,1	6,1
NA	36,6	30,6	6

Multiple linear regression models for each group.

Group	N	Formula	R2	Adjusted_R2	F	df1	df2	Model_p
aMCI	11	EF ~ BOA + PE + NTE	0,748	0,640	6,917	3	7	0,017
Healthy	12	EF ~ BOA + PE	0,159	-0,028	0,848	2	9	0,460

*The degrees of freedom are: df1 = selected predictors; df2 = N-k-1 (N = sample size; K = selected predictors; 1 = intercept).

The table shows the effect of predictors that contributed significantly to the model in relation to EF as a criterion in the aMCI group.

Variable	Estimate	Std..Error	CI_lower	CI_upper	t.value	*p*
(Intercept)	252,648	54,056	6,984	347,657	4,674	0,002
BOA	-4,865	1,742	-8,930	1,462	-2,792	0,027
PE	-1,512	0,370	-2,232	0,259	-4,089	0,005
NTE	0,082	0,159	-0,640	0,662	0,516	0,622

The table shows the effect of predictors that contributed significantly to the model in relation to EF as a criterion in the Healthy group.

Variable	Estimate	Std..Error	CI_lower	CI_upper	t.value	*p*
(Intercept)	68,393	21,436	28,475	151,133	3,191	0,011
BOA	-1,248	1,491	-5,004	1,356	-0,837	0,424
PE	0,134	0,131	-0,671	0,383	1,023	0,333

Role Limitations Due to Physical Health **(RLDPH)**

Predictor selection based on bootstrap-enhanced LASSO procedure. Predictor>50%.

Predictor	aMCI_percent	Healthy_percent	Difference
NTE	65,33	27,03	38,30
PE	94,69	58,36	36,33
NE	49,70	41,64	8,06
PA	41,38	34,43	6,95
NTA	33,87	28,03	5,84
NA	36,27	39,14	-2,87

Multiple linear regression models for each group.

Group	N	Formula	R2	Adjusted_R2	F	df1	df2	Model_p
aMCI	11	LRDSF ~ BOA + PE + NTE	0,765	0,665	7,614	3	7	0,01
Healthy	12	LRDSF ~ BOA + PE	0,303	0,148	1,956	2	9	0,19

*The degrees of freedom are: df1 = selected predictors; df2 = N-k-1 (N = sample size; K = selected predictors; 1 = intercept).

The table shows the effect of predictors that contributed significantly to the model in relation to RLDPH as a criterion in the aMCI group.

Variable	Estimate	Std..Error	CI_lower	CI_upper	t.value	*p*
(Intercept)	221,609	101,917	-19,387	462,604	2,174	0,06
BOA	-2,266	3,285	-10,034	5,501	-0,690	0,51
PE	-2,047	0,697	-3,696	-0,398	-2,936	0,02
NTE	0,450	0,300	-0,259	1,159	1,502	0,17

The table shows the effect of predictors that contributed significantly to the model in relation to RLDPH as a criterion in the Healthy group.

Variable	Estimate	Std..Error	CI_lower	CI_upper	t.value	*p*
(Intercept)	116,719	65,461	-31,363	264,802	1,783	0,108
BOA	-6,726	4,554	-17,026	3,575	-1,477	0,174
PE	0,543	0,399	-0,360	1,445	1,361	0,207

Limitations Due to Emotional Health (**LDEH**)

Predictor selection based on bootstrap-enhanced LASSO procedure. Predictor>50%.

Predictor	aMCI_percent	Healthy_percent	Difference
PE	65,63	35,93	29,70
NA	28,84	46,79	-17,95
NTE	33,77	28,52	5,25
NE	31,56	36,79	-5,23
NTA	41,81	37,41	4,40
PA	34,37	38,64	-4,27

Multiple linear regression models for each group.

Group	N	Formula	R2	Adjusted_R2	F	df1	df2	Model_p
aMCI	11	LRDPE ~ BOA + PE	0,432	0,289	3,037	2	8	0,10
Healthy	12	LRDPE ~ BOA	0,028	-0,070	0,285	1	10	0,60

*The degrees of freedom are: df1 = selected predictors; df2 = N-k-1 (N = sample size; K = selected predictors; 1 = intercept).

The table shows the effect of predictors that contributed significantly to the model in relation to RLDPH as a criterion in the aMCI group.

Variable	Estimate	Std..Error	CI_lower	CI_upper	t.value	*p*
(Intercept)	297,418	162,851	-78,116	672,952	1,826	0,10
BOA	-3,318	5,575	-16,173	9,537	-0,595	0,56
PE	-2,360	1,123	-4,949	0,229	-2,102	0,06

The table shows the effect of predictors that contributed significantly to the model in relation to RLDPH as a criterion in the Healthy group.

Variable	Estimate	Std..Error	CI_lower	CI_upper	t.value	*p*
(Intercept)	114,895	49,311	5,023	224,766	2,330	0,04
BOA	-2,012	3,771	-10,415	6,391	-0,534	0,60

Social Functioning (**SF**)

Predictor selection based on bootstrap-enhanced LASSO procedure. Predictor>50%.

Predictor	aMCI_percent	Healthy_percent	Difference
NTA	55,68	27,90	27,78
PE	70,15	47,90	22,25
PA	55,28	33,10	22,18
NTE	44,32	31,90	12,42
NA	41,81	36,10	5,71
NE	35,48	35,20	0,28

Multiple linear regression models for each group.

Group	N	Formula	R2	Adjusted_R2	F	df1	df2	Model_p
aMCI	11	FS ~ BOA + PE + NTA + PA	0,643	0,405	2,700	4	6	0,13
Healthy	12	FS ~ BOA	0,146	0,060	1,705	1	10	0,22

*The degrees of freedom are: df1 = selected predictors; df2 = N-k-1 (N = sample size; K = selected predictors; 1 = intercept).

The table shows the effect of predictors that contributed significantly to the model in relation to RLDPH as a criterion in the aMCI group.

Variable	Estimate	Std..Error	CI_lower	CI_upper	t.value	*p*
(Intercept)	254,305	162,390	-143,050	651,659	1,566	0,168
BOA	-3,199	5,527	-16,723	10,326	-0,579	0,584
PE	-1,848	0,965	-4,209	0,512	-1,916	0,104
NTA	-0,398	0,354	-1,265	0,469	-1,124	0,304
PA	0,523	0,557	-0,840	1,886	0,939	0,384

The table shows the effect of predictors that contributed significantly to the model in relation to RLDPH as a criterion in the Healthy group.

Variable	Estimate	Std..Error	CI_lower	CI_upper	t.value	*p*
(Intercept)	98,541	20,895	51,983	145,099	4,716	0,001
BOA	-2,087	1,598	-5,647	1,474	-1,306	0,221

Pain (**P**)

Predictor selection based on bootstrap-enhanced LASSO procedure. Predictor>50%.

Predictor	aMCI_percent	Healthy_percent	Difference
NTE	39,45	61,40	-21,95
NA	26,50	46,70	-20,20
PA	32,62	49,80	-17,18
PE	51,07	59,00	-7,93
NE	29,56	36,60	-7,04
NTA	46,59	43,30	3,29

Multiple linear regression models for each group.

Group	N	Formula	R2	Adjusted_R2	F	df1	df2	Model_p
aMCI	11	D ~ BOA + PE	0,387	0,234	2,527	2	8	0,141
Healthy	12	D ~ BOA + NTE + PE	0,347	0,102	1,415	3	8	0,308

*The degrees of freedom are: df1 = selected predictors; df2 = N-k-1 (N = sample size; K = selected predictors; 1 = intercept).

The table shows the effect of predictors that contributed significantly to the model in relation to RLDPH as a criterion in the aMCI group.

Variable	Estimate	Std..Error	CI_lower	CI_upper	t.value	*p*
(Intercept)	301,473	123,370	16,983	585,964	2,444	0,040
BOA	-5,136	4,223	-14,875	4,602	-1,216	0,259
PE	-1,865	0,850	-3,826	0,096	-2,193	0,060

The table shows the effect of predictors that contributed significantly to the model in relation to RLDPH as a criterion in the Healthy group.

Variable	Estimate	Std..Error	CI_lower	CI_upper	t.value	*p*
(Intercept)	44,498	21,972	-6,170	95,166	2,025	0,077
BOA	-0,505	1,492	-3,946	2,936	-0,338	0,744
NTE	0,300	0,188	-0,133	0,733	1,596	0,149
PE	0,154	0,123	-0,130	0,438	1,249	0,247

General Health (**GH**)

Predictor selection based on bootstrap-enhanced LASSO procedure. Predictor>50%.

Predictor	aMCI_percent	Healthy_percent	Difference
PA	64,8	37,9	26,9
PE	80,3	66,4	13,9
NE	39,8	52,7	-12,9
NTE	47	59,9	-12,9
NTA	46,3	34,9	11,4
NA	31	30,3	0,7

Multiple linear regression models for each group.

Group	N	Formula	R2	Adjusted_R2	F	df1	df2	Model_p
aMCI	11	SG ~ BOA + PE + PA	0,781	0,687	8,306	3	7	0,010
Healthy	12	SG ~ BOA + PE + NTE + NE	0,703	0,533	4,135	4	7	0,050

*The degrees of freedom are: df1 = selected predictors; df2 = N-k-1 (N = sample size; K = selected predictors; 1 = intercept).

The table shows the effect of predictors that contributed significantly to the model in relation to RLDPH as a criterion in the aMCI group.

Variable	Estimate	Std..Error	CI_lower	CI_upper	t.value	*p*
(Intercept)	210,700	61,407	29,312	387,278	3,431	0,011
BOA	-3,819	2,087	-10,117	2,500	-1,830	0,110
PE	-1,513	0,344	-2,311	-0,635	-4,392	0,003
PA	0,202	0,211	-0,714	0,636	0,957	0,370

The table shows the effect of predictors that contributed significantly to the model in relation to RLDPH as a criterion in the Healthy group.

Variable	Estimate	Std..Error	CI_lower	CI_upper	t.value	*p*
(Intercept)	71,069	24,518	20,177	164,209	2,899	0,023
BOA	-3,819	1,324	-8,890	-0,213	-2,884	0,024
PE	0,273	0,136	-0,140	0,990	2,009	0,085
NTE	0,279	0,213	-0,434	0,718	1,307	0,233
NE	-0,183	0,170	-1,047	0,125	-1,077	0,317
